# Eco-Friendly Carbon Nanotubes Reinforced with Sodium Alginate/Polyacrylic Acid for Enhanced Adsorption of Copper Ions: Kinetics, Isotherm, and Mechanism Adsorption Studies

**DOI:** 10.3390/molecules29194518

**Published:** 2024-09-24

**Authors:** Pengbo Chang, Shuyang Zhou, Tongchao Wang, Dangling Hua, Shiliang Liu, Oseweuba Valentine Okoro, Armin Shavandi, Lei Nie

**Affiliations:** 1 College of Resources and Environment, Henan Agricultural University, Zhengzhou 450046, China; cpb317@163.com (P.C.); collegehua@163.com (D.H.); shlliu70@163.com (S.L.); 2 Zhengzhou Technical College, Zhengzhou 450121, China; 3 College of Life Sciences, Xinyang Normal University, Xinyang 464000, China; zhoushuyang18@163.com; 4 College of Agronomy, Henan Agricultural University, Zhengzhou 450046, China; 5 3BIO-BioMatter, École Polytechnique de Bruxelles, Université Libre de Bruxelles (ULB), Avenue F.D. Roosevelt, 50-CP 165/61, 1050 Brussels, Belgium

**Keywords:** hydrogel, carbon nanotube, copper, adsorption kinetics, adsorption isotherms

## Abstract

This study investigates the removal efficiency of Cu^2+^ from wastewater using a composite hydrogel made of carbon nanotubes (CNTs), sodium alginate (SA), and polyacrylic acid (PAA) prepared by free radical polymerization. The CNTs@SA/PAA hydrogel’s structure and properties were characterized using SEM, TEM, FTIR, XRD, rheology, DSC, EDS, elemental mapping analysis, and swelling. The adsorption performance for Cu^2+^ was tested in batch adsorption experiments, considering the pH, dosage, initial concentration, and contact time. The optimal conditions for Cu^2+^ removal were pH 5.0, an adsorbent dosage of 500 mg/L, and a contact time of 360 min. The adsorption followed pseudo-second order kinetics. Isotherm analyses (Langmuir, Freundlich, Temkin, Dubinin–Radushkevich, Sips, Toth, and Khan) revealed that the Freundlich isotherm best described the adsorption, with a maximum capacity of 358.52 mg/g. A thermodynamic analysis indicated that physical adsorption was the main interaction, with the spontaneity of the process also demonstrated. This study highlights the high efficiency and environmental friendliness of CNT@SA/PAA composites for Cu^2+^ removal from wastewater, offering a promising approach for water treatment.

## 1. Introduction

In recent years, human activities and the development of industry and agriculture have increased environmental pollution, leading to the contamination of soil and water with heavy metal ions [1]. Water pollution has become a major environmental challenge [2,3,4,5], posing a threat to the health of humans and other organisms and offsetting the balance of aquatic ecosystems [6,7,8,9,10]. Given that heavy metals are typically toxic, bioaccumulative, and persistent, many researchers have focused on eliminating heavy metal pollution with an emphasis on the removal of copper ions [11,12,13]. Copper is one of the most important trace elements that influences metabolic processes. Thus, the guidelines of the World Health Organization and the US Environmental Protection Agency recommend limits of 2.0 mg/L and 1.3 mg/L, respectively, for copper in drinking water [14,15]. However, relatively higher concentrations of copper ions could lead to diseases in humans, such as cancer and Wilson’s disease, and affect the body’s immune and nervous systems [16,17,18,19,20,21]. Due to these potential negative implications of higher copper ion concentrations on humans, it is necessary to remove the excess potentially toxic copper ions from water and wastewater, improving the water quality.

Until now, different technologies have been developed for the treatment of heavy metal copper water pollution, such as chemical precipitation, ion exchange, membrane separation, the electrochemical method, electrostatic attraction, solvent extraction, reverse osmosis, flocculation, and adsorption [22,23,24,25,26,27,28]. The adsorption method presents a prominent prospect in the removal of heavy metal due to its high removal efficiencies, low secondary pollution, low cost, and numerous potential adsorbents [29,30,31,32,33,34,35]. The adsorbent material plays an important role in the adsorption efficiency of minerals (montmorillonite, kaolin, bentonite, and zeolite) [36,37,38,39], carbon adsorption materials (biochar, carbon nanotubes, and graphene) [40], adsorption materials of metal compounds (MOFs) [41], and gels (sodium alginate, chitosan, gelatin, starch, cellulose, pectin, agarose, and xanthan gum) [42], etc. Among these materials, gel adsorption materials based on natural polymers are predominantly chosen due to their excellent biocompatibility, natural degradation, high stability, and ability to facilitate the easy formation of a 3-D network structure [43]. The hydrogels’ outstanding 3-D network structure and substantial specific surface area significantly enhance the potentiality for interaction with heavy metal ions, thereby improving their efficiencies in removing such ions, leading to wide applications [44,45,46]. However, this kind of hydrogel suffers from some drawbacks, such as chemical instability, less exposed active sites, and limited capability to adsorb heavy metal ions. The incorporation of certain carbon-based adsorbents with the hydrogel could increase the adsorption efficiency and enhance the mechanical characteristics.

Sodium alginate (SA, C_6_H_7_NaO_6_) is a natural hydrophilic anionic biopolymer extracted from brown algae kelp or sargassum. SA is a copolymer composed of β-d-mannuronic acid (M unit) and α-l-guluronic acid (G unit) linked by β-1,4-glycosidic bonds [47]. The SA surface is rich in hydroxyl and carboxyl groups, typically forming rigid and compact gels when interacting with divalent alkaline earth metal ions, such as Ca^2+^ and Ba^2+^, via a salt bridge. This characteristic results in a robust binding capacity for heavy metal ions [48]. However, the instability, weak mechanical strength, and low adsorption capacity of SA gel limit the polymer’s application [49]. Hence, SA has been frequently combined with other adsorption materials to prepare composite hydrogels, enhancing the adsorption capacity for heavy metal ions. A combination of SA, magnetic chitosan, and polyethyleneimine was employed to prepare a composite gel that could effectively remove heavy metal ions and azo dyes from water [50].

Polyacrylic acid (PAA) is a polymer formed by polymerization of an acrylic acid monomer (AA) [51,52,53], with the characteristics of good water solubility, abundant carboxyl groups, and excellent water absorption capability. It is easy for PAA to form a 3-D network structure in aqueous solutions, which allows the release of anionic functional groups (–COO^−^), enabling the chelation of positively charged heavy metal ions, thus removing heavy metal ions from aqueous solutions [54,55].

Carbon nanotubes (CNTs) are carbon adsorbents with unique characteristics of thermal stability, thermal conductivity, catalysis, and adsorption capability [56]. There are single-walled (SWCNTs) and multi-walled (MWCNTs) nanotubes [57]. Due to the nanoscale structures, CNTs have a very large surface area of active sites and thus could adsorb heavy metal ions to the surface through hydrogen bonding, electrostatic attraction, and van der Waals forces. Additionally, CNTs could be chemically bonded to heavy metal ions with carboxyl or hydroxyl groups, increasing the adsorption efficiency and capability. As for the applications to wastewater treatment, CNTs have shown excellent adsorption selectivity [58]. However, the high costs and difficulties in recycling CNTs limit their applications in water and wastewater treatments, which could be solved via their integration into natural polymer networks [59,60]. 

**Scheme 1:**
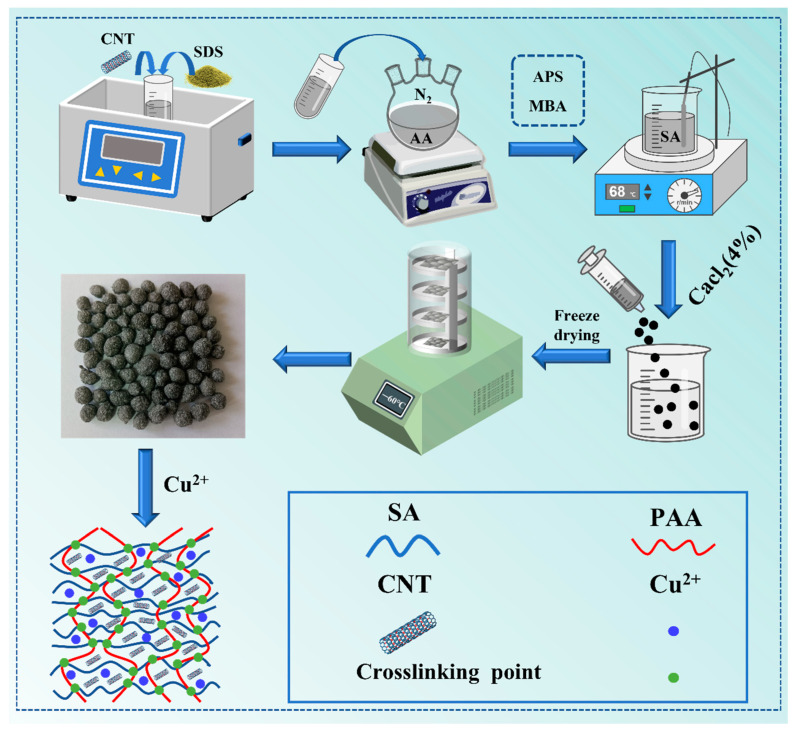
The preparation process of CNTs@SA/PAA hydrogel.

In this study, a novel composite hydrogel adsorbent composed of SA, PAA, and CNTs, CNTs@SA/PAA, was prepared with free radical polymerization, as shown in Scheme 1, using *N*,*N*’-methylene bisacrylamide (MBA) and ammonium persulfate (APS) as the crosslinking agent and initiator, respectively. The resultant CNTs@SA/PAA hydrogel adsorbents were characterized with a scanning electron microscope–energy dispersive spectrometer (SEMEDS), X-ray diffraction (XRD), Fourier transform infrared spectrometer (FTIR), rheology and differential scanning calorimetry (DSC), etc. The adsorption capacities of the resultant hydrogels were investigated using the static adsorptions of heavy metal ions. The effects of different conditions on the adsorption capacity of the CNTs@SA/PAA hydrogels were investigated, including the pH of an aqueous solution, the adsorbent dosage, the contact time, and the initial Cu^2+^ concentration. The adsorption kinetics, behavior, and capacity of the CNTs@SA/PAA hydrogels were analyzed with kinetic and isothermal models, and their adsorption mechanisms for the removal of copper ions are discussed extensively.

## 2. Results and Discussion

### 2.1. Characterizations

#### 2.1.1. Two-Dimensional Morphologies

**Figure 1:**
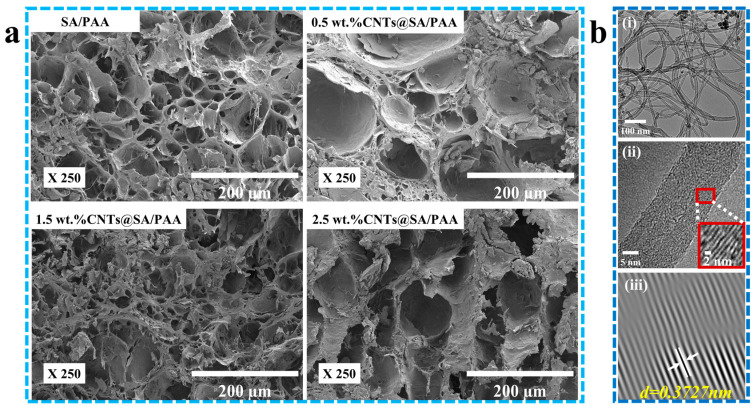
(**a**) SEM images of SA/PAA, 0.5 wt.%CNTs@SA/PAA, 1.5 wt.%CNTs@SA/PAA, and 2.5 wt.%CNTs@SA/PAA. (**b**) TEM image of CNTs.

Figure 1 shows the 2-D images of the resultant hydrogels, SA/PAA, 0.5 wt.%CNTs@SA/PAA, 1.5 wt.%CNTs@SA/PAA, and 2.5 wt.%CNTs@SA/PAA, observed with SEM, and the images of the CNTs visualized with TEM. It can be seen from Figure 1a that the surface of the SA/PAA hydrogel is flat and smooth [61,62]. Figure 1b shows that there are numerous irregular pores with different sizes in all the CNTs@SA/PAA composite gels, which could be beneficial to the adsorption process. The laminated structure of the CNTs (d = 0.3727 nm) could be seen clearly in the TEM images of the CNTs. The composite hydrogels were prepared via doping the CNTs with the lamellar structure into the SA/PAA hydrogel with the porous structure and show a honeycomb shape, suggesting that the CNTs have been dispersed into the main body of the SA/PAA hydrogels. 

#### 2.1.2. Chemical Structure of the Resultant Composite Hydrogels 

**Figure 2:**
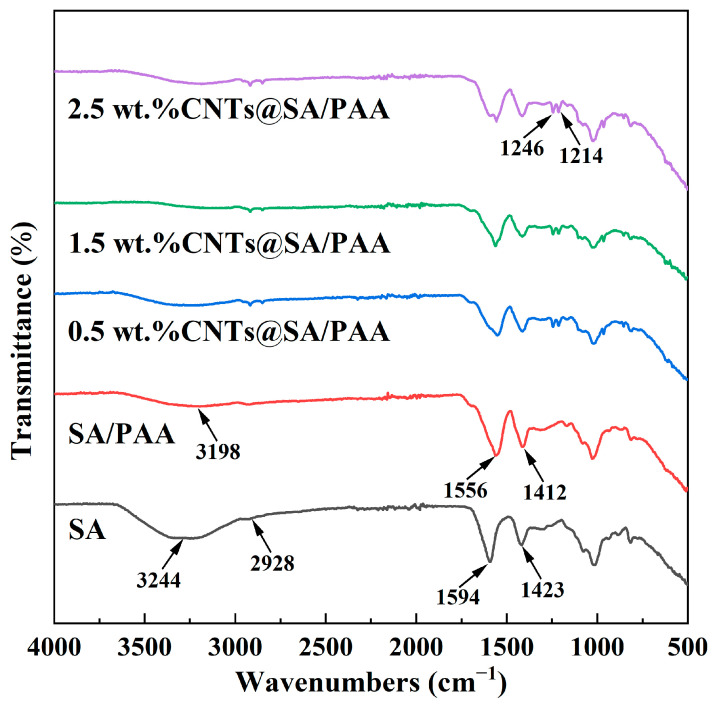
FT-IR spectra of SA, SA/PAA, and CNTs@SA/PAA composite hydrogels.

Figure 2 shows the FTIR spectra of the SA, SA/PAA, 0.5 wt.%CNTs@SA/PAA, 1.5 wt.%CNTs@SA/PAA, and 2.5 wt.%CNTs@SA/PAA hydrogels. As for SA, the stretching vibration of the characteristic –OH functional group was observed at the peaks of 3244 cm^−1^ and 2928 cm^−1^, respectively. The symmetrical and asymmetric vibration spectra peaks of the –COO–functionality groups were observed at 1594 cm^−1^ and 1423 cm^−1^, respectively [63]. As for the SA grafted with PAA, the wide adsorption band at 3198 cm^−1^ could be attributed to the superimposition of the vibrations of –OH in SA and PAA. The adsorption bands at 1556 cm^−1^ and 1412 cm^−1^ are attributed to the symmetrical and asymmetrical vibration spectra peaks of the –COO– groups. The characteristic peak of C=C disappeared in the SA grafted with PAA, indicating the occurrence of a successfully grafted copolymerization [64]. The position and strength of the peaks of the O-containing groups significantly changed as the CNTs were combined with SA/PAA. As seen clearly from Figure 2, a weak broad peak in the range of 3100–3700 cm^−1^, attributed to the hydroxyl (–OH) groups, appeared in the FTIR spectra of all the CNTs@SA/PAA composite hydrogels. Compared with the SA and SA/PAA hydrogels, there are two new peaks shown in the spectra of the CNTs@SA/PAA hydrogels, 1246 cm^−1^ and 1214 cm^−1^, attributed to the stretching vibration of the O–H bonds of the carboxyl groups. Hence, the FTIR spectra suggest that both the SA and CNTs reacted with the acrylic monomers during the polymerization and a new kind of polymer network was prepared in the CNTs@SA/PAA composite hydrogels.

#### 2.1.3. Differential Scanning Calorimetry (DSC)

**Figure 3:**
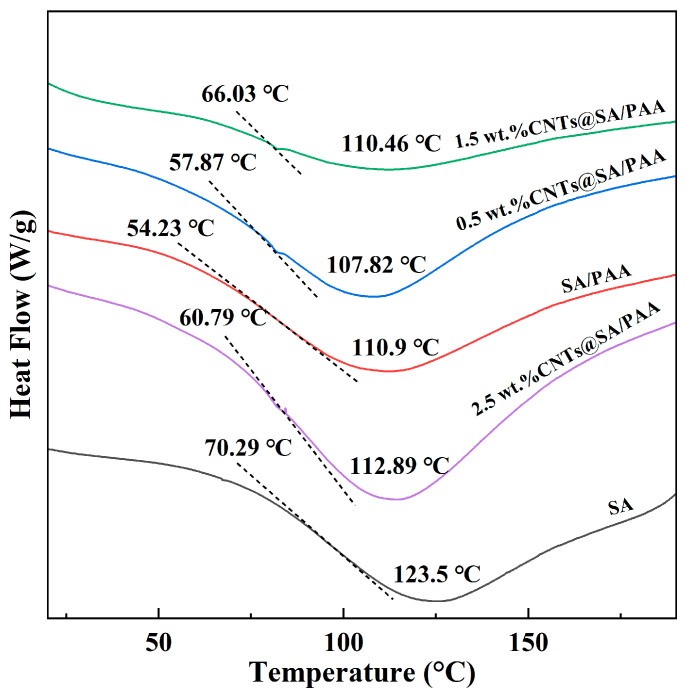
DSC scan of SA, SA/PAA, and CNTs@SA/PAA composite hydrogels: heat flow vs. temperature.

Figure 3 shows the DSC results of the resultant SA, SA/PAA, and CNTs@SA/PAA hydrogels. It could be known from the figure that the *T_g_* of the SA/PAA hydrogel is around 54.2 °C. This suggests that the doping of the CNTs could increase the *T_g_* of the resultant composite hydrogels. The T_g_ of the 0.5 wt.%CNTs@SA/PAA, 1.5 wt.%CNTs@SA/PAA, and 2.5 wt.%CNTs@SA/PAA were 57.8 °C, 66.0 °C, 60.7 °C, respectively, which might be due to the limitation of the movement of the polymer chains caused by the insertion of nanosheets into the polymer networks. The temperature of the melting peak of the CNTs@SA/PAA hydrogel is lower than that of the SA/PAA, which might be due to the acceleration of heat absorption by the thermal conductive nano-fillers. As the amount of CNTs is in a certain range of 0–1.5 wt.%, the temperature of the melting peak is lower than that of the polymer hydrogel without doping of the CNTs. However, the excessive addition of CNTs, 2.5 wt.% herein, will increase the melting peak’s temperature [65].

#### 2.1.4. XRD Analysis

**Figure 4:**
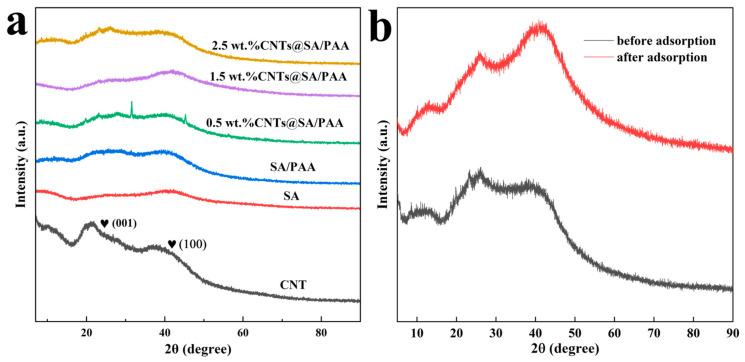
(**a**) X-ray diffraction patterns of SA, SA/PAA, and CNTs@SA/PAA hydrogels, and (**b**) comparison of 1.5 wt.%CNTs@SA/PAA hydrogel before and after Cu^2+^ adsorption.

The XRD patterns of the CNTs, SA, SA/PAA, and CNTs@SA/PAA are shown in Figure 4a. It could be seen clearly that the characteristic diffraction peaks of the CNTs at 2θ were 26° and 42°, corresponding to the (001) and (100) crystal planes of the cubic CNTs, respectively, which were significantly weaker in all the XRD patterns of the CNTs@SA/PAA composite hydrogels [66]. The reason might be the combination of CNTs with SA and PAA.

Figure 4b shows the contrast diagram of the 1.5 wt.%CNTs@SA/PAA composite hydrogels before and after the adsorptions of copper ions using the composite hydrogels. After the adsorption of Cu^2+^ ions, more substantial peaks appeared at about 25.9° and 41.8°, indicating the reaction between the Cu^2+^ ions and the CNTs@SA/PAA composite hydrogels and the enhancement of the crystal structures.

#### 2.1.5. Rheological Analysis 

**Figure 5:**
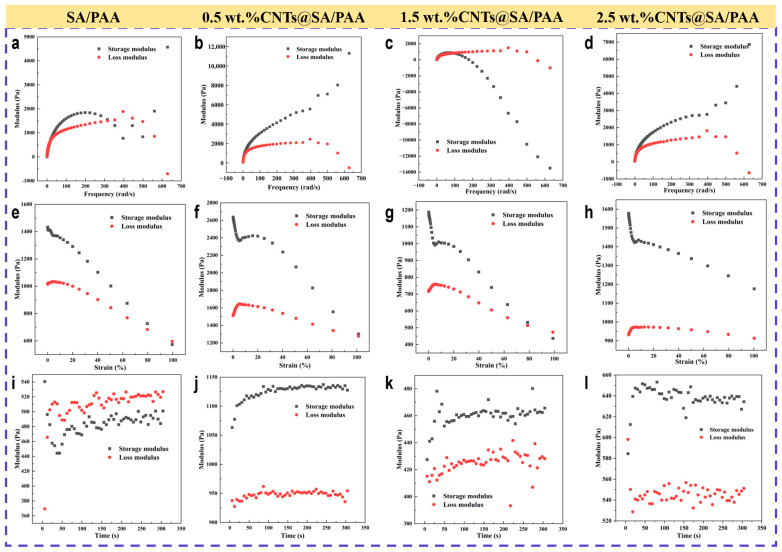
Rheological properties of SA/PAA hydrogel and CNTs@SA/PAA composite hydrogels with different CNT weight ratios: (**a**–**d**). *G*′ and *G*″ vs. frequency, (**e**–**h**): *G*′ and *G*″ vs. strain, and (**i**–**l**): *G*′ and *G*″ vs. time.

Figure 5 shows the rheological properties of the resultant hydrogels, which were the typical hydrogel behaviors, i.e., the storage modulus (*G*′) consistently surpassed the loss modulus (*G*″). The *G*″ of almost all the hydrogels except 1.5 wt.%CNTs@SA/PAA increased with the increasing shear frequency. 

*G*′ and *G*″ converged gradually, suggesting the breakdown of the hydrogel structure. Despite this convergence, *G*′ continued to dominate over *G*″, highlighting the hydrogels’ solid-state nature and elastic deformation. As seen clearly from Figure 5i–l, the G′ of the CNTs@SA/PAA hydrogel was always higher than the G″, suggesting that the mechanical properties of the resultant composite hydrogels doped with CNTs were better than of those of the pure SA and SA/PAA.

#### 2.1.6. EDS and Elemental Mapping Analysis

**Figure 6:**
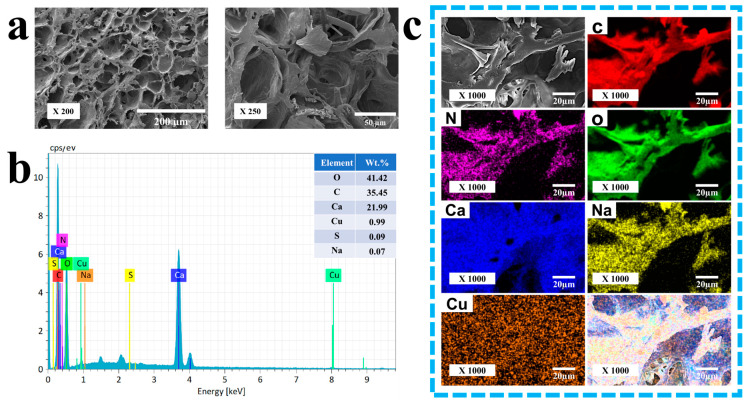
Characterizations of 1.5 wt.%CNTs@SA/PAA hydrogel with EDS: (**a**) SEM images of the hydrogel porous structure after the adsorption of Cu^2+^ ions, (**b**) EDS spectrum of 1.5 wt.%CNTs@SA/PAA-Cu, (**c**) EDS elemental mapping of 1.5 wt.%CNTs@SA/PAA hydrogel after Cu^2+^ ion adsorption.

EDS elemental mapping was used to further elucidate the absorption process of Cu^2+^ ions on the CNTs@SA/PAA composite hydrogels. Figure 6a shows the SEM images of CNTs@SA/PAA-Cu. It could be seen clearly that the structure of the CNTs@SA/PAA composite hydrogels was still layered and porous, resulting in a higher adsorption capacity and efficiency. Figure 6b,c shows the element mapping results and the energy dispersive X-ray (EDX) spectrum. It is suggested that the hydrogel, after absorbing Cu^2+^ ions, contains C, H, O, Ca, Na, Cu, etc. As seen clearly from Figure 6c, the distribution of Cu throughout the adsorbent, 1.5 wt.%CNTs@SA/PAA, was uniform and dense, suggesting its excellent mechanical stability [65,67]. In addition, the presence of calcium ions was also found in Figure 6c, indicating that calcium ions were also involved in the synthesis of the hydrogels. 

### 2.2. Adsorption Batch Experiment

#### 2.2.1. Swelling Behaviors of the Resultant Hydrogels 

**Figure 7:**
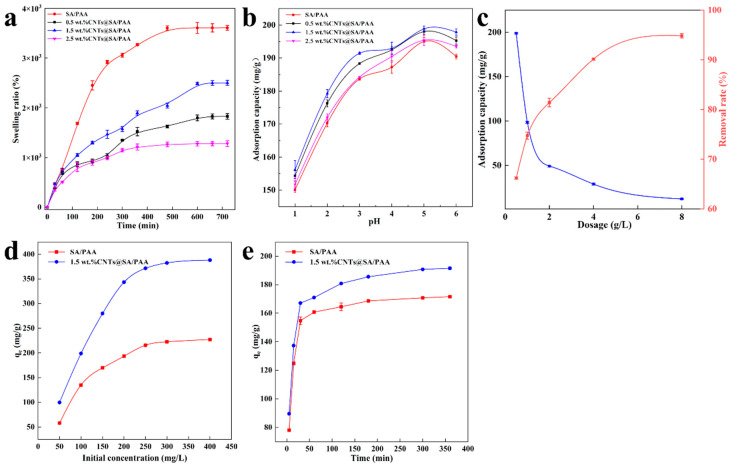
(**a**) Swelling ratio of the resultant hydrogels vs. time, (**b**) adsorption capacities at different initial pH values, (**c**) adsorption capacity and removal ratio vs. the dosage of 1.5 wt.%CNTs@SA/PAA hydrogel, (**d**) *q_e_* vs. initial Cu^2+^ concentration, and (**e**) *q_e_* vs. contact time.

The swelling behaviors vs. time of SA/PAA and CNTs@SA/PAA are shown in Figure 7a. It was found that the swelling ratio of all the resultant hydrogels increased sharply in the initial 12 h after immersion in UPW, and the swelling ratios of the hydrogels doped with CNTs are lower than those of the SA/PAA hydrogels. The more CNTs doped, the lower the swelling ratio of the resultant hydrogels, which might be due to the hydrophobicity of CNTs.

#### 2.2.2. Effect of pH on the Adsorption Capacity of the Resultant Hydrogels 

The pH value of the aqueous solution has a significant effect on the adsorption capacity of the resultant hydrogels because, due to the surface charge of the hydrogels, the form of Cu^2+^ in the solution is different at different pH levels [68]. Figure 7b shows the adsorption capacities of the hydrogels for Cu^2+^ at different pHs ranging from 1.0 to 6.0. As can be clearly seen, the adsorption capacities of the hydrogels for Cu^2+^ increased with the increasing pH in the range of 1 to 5, and then decreased with the increasing pH. As the pH was in the range of 1 to 5, the electrostatic repulsions between the protonated functional groups on the hydrogel surface and the Cu^2+^ inhibited the adsorption. On the other hand, the competition between Cu^2+^ and H^+^ to chelate with the functional groups of hydrogels decreases the adsorption capacity, as well. The adsorption capacity of 1.5 wt.%CNTs@SA/PAA for Cu^2+^ reached the maximum at pH 5, 198.84 mg/g. The 1.5 wt.%CNTs@SA/PAA hydrogel and pH 5 were used for the following investigations [69,70].

#### 2.2.3. Effect of the Adsorbent Dosage

Figure 7c shows the effect of the 1.5 wt.%CNTs@SA/PAA dosage on Cu^2+^ adsorption at pH 5. The removal rate of Cu^2^ increased as the adsorbent dosage increased, while the adsorption capacity decreased. The reason for this is that the number of active sites for adsorption increases with the increase in dosage. The adsorption capacity gradually leveled off as the dosage of 1.5 wt.%CNTs@SA/PAA was higher than 100 mg. In fact, the higher the dosage, the lower the adsorption capacity of Cu^2+^. This can be attributed to the presence of unsaturated adsorption sites during the adsorption reaction. The decrease in accessible adsorption sites may be due to the aggregation of adsorbent molecules caused by surface interaction. Finally, an equilibrium is reached between the adsorbed Cu^2+^ and the unadsorbed Cu^2+^ in the solution [71]. Therefore, from an economic point of view, the 25 mg hydrogel was chosen for subsequent experiments.

#### 2.2.4. Effects of the Initial Pollutant Concentration

Figure 7d shows that the adsorption capacity gradually increased with the increase of the initial concentration of Cu^2+^. The reason for this might be that the sites of the adsorbent are not completely occupied at the low Cu^2+^ concentrations. As the Cu^2+^ concentration was higher than 300 mg/L, all the free sites of the 1.5 wt.%CNTs@SA/PAA hydrogels were saturated gradually, and the adsorption equilibrium was reached. There were more adsorption sites for adsorbing Cu^2+^ due to the doping of the CNTs [72]. Thus, the adsorption capacity of 1.5 wt.%CNTs@SA/PAA was 71% higher than that of the SA/PAA hydrogel.

#### 2.2.5. Effects of the Contact Time

Figure 7e shows the effects of the contact time on Cu^2+^ adsorption. The adsorption capacity of Cu^2+^ increased rapidly and then gradually slowed down with the increase in the contact time, until the adsorption equilibrium reached 360 min. In the early stage, the 1.5 wt.%CNTs@SA/PAA hydrogel had numerous free adsorption sites and a high specific surface area, resulting in a higher probability of contact between Cu^2+^ and the binding sites. As the contact went by, Cu^2+^ chelated with the functional groups and approached the active sites of the hydrogels and the CNTs until saturation was achieved [73,74].

#### 2.2.6. Adsorption Kinetics Analysis

**Figure 8:**
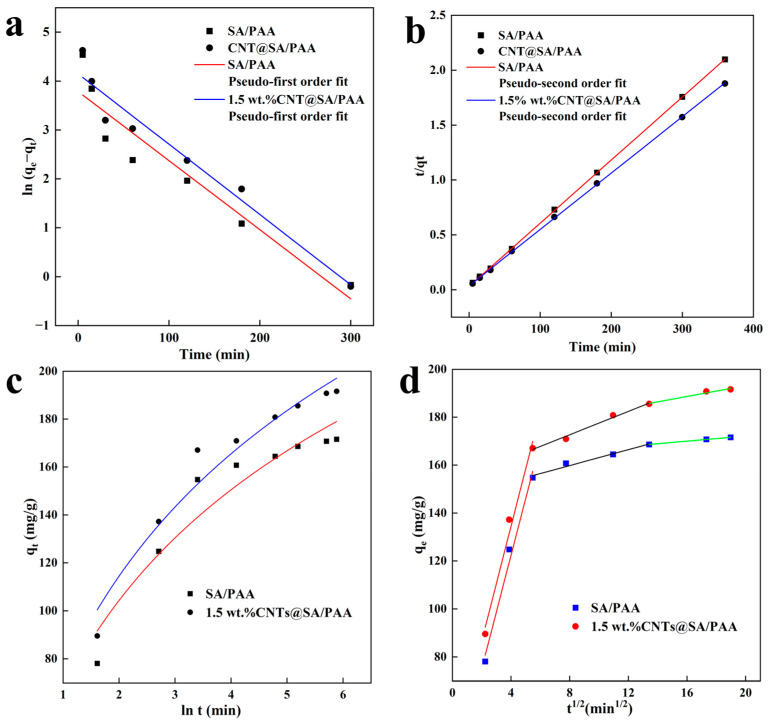
(**a**) Adsorption kinetics of Cu^2+^ onto the resultant composite hydrogels, the kinetic plots of pseudo-first order and (**b**) pseudo-second order kinetic fitting curves. (**c**) Elovich and (**d**) intraparticle diffusion models for Cu^2+^ adsorption. Adsorption conditions: 1.5 wt.%CNTs@SA/PAA hydrogel, adsorbent dosage 500 mg/L, pH 5, and temperature 25 °C.

Figure 8 shows the results of the experimental fitting data. Table 1 shows the kinetic parameters based on Figure 8a,b.

Table 1 shows that the *R*^2^ of the pseudo-second order kinetic model of the two adsorbents, SA/PAA and 1.5 wt.%CNT@SA/PAA, was greater than 0.9998, almost close to 1, indicating relatively high fitting degrees. In addition, the calculated *q_e_* values of adsorbents SA/PAA and 1.5 wt.%CNT@SA/PAA were 173.913 mg/g and 194.553 mg/g, respectively, which were closer to the actual experimental adsorption capacity *q_e_* (171.57 mg/g and 191.57 mg/g). Therefore, the pseudo-second order model is more conducive to describe the adsorption process of copper ions on adsorbents SA/PAA and 1.5 wt.%CNT@SA/PAA. However, the *R*^2^ of the pseudo-first order kinetic model of the two hydrogels was also higher than 0.88, which also describes the adsorption process of Cu^2+^ better. Consequently, the adsorption processes of Cu^2+^ by the SA/PAA and 1.5 wt.%CNTs@SA/PAA adsorbents might be the combination of chemical adsorption and physical adsorption, including pore filling and electrostatic adsorption.

Hence, adjusting the reaction temperature and pH, commonly employed to enhance the rate of the chemisorption process, can serve as the primary approach to improve their adsorption efficiency. Furthermore, the experimental adsorption capacity (*q_e_*) of the two adsorbents for Cu^2+^ closely matches the calculated values, indicating a good fit of the data with the model. 

The Elovich model was employed to study the adsorption mechanism of Cu^2+^ because of the non-homogeneous nature of the adsorbent surface and the diversity of the adsorption process. The equation of the Elovich model is based on the kinetic principle and is used to describe the adsorption of heterogeneous adsorbents. It assumes that the adsorption sites increase exponentially with the adsorption, and multilayer adsorption might occur. The experimental constants *α* and *β* are calculated from the slopes and intercepts in the nonlinear plots of *q_e_* versus ln *t* (Figure 8c). Table 2 shows the fitting of the kinetic data with the Elocvich model.

It could be known from Table 2 that the correlation coefficients of both adsorbents for Cu^2+^ ions adsorptions were higher than 0.9. The adsorption behaviors followed the Elovich kinetic model, suggesting that the chemisorption mechanism is the dominant mechanism governing the adsorption of Cu^2+^ ions, which is consistent with the kinetic results in Table 1 and highlights that a relatively high *R*^2^ value was present due to the dominance of the chemisorption mechanism. 

The *α* value of the CNTs@SA/PAA hydrogel is approximately 9% higher than that of the SA/PAA hydrogel, while the *β* value is about 10% lower than that of the SA/PAA hydrogel. The reason for this might be the decrease in the activation energy due to the doping of the CNTs, which is favorable for adsorption. 

Figure 8d shows a plot of *q_t_* vs. *t*^0.5^ for adsorption with the values of *C* and *k_id_* presented in Table 3, where the intraparticle diffusion model proposed by Weber and Morris has been widely applied for the analysis of adsorption kinetics at the solid–liquid interface. As clearly seen from Figure 8d, the plot of *q_t_* vs. *t*^0.5^ shows multi-segment linear characteristics, indicating that the adsorption process might be controlled by multiple mechanisms [75]. The plot based on the intraparticle diffusion model shows three distinct stages. As known from Figure 8d and Table 3, the migration of Cu^2+^ ions from the aqueous solution to the surface interface of the adsorbent occurred in the first stage. The rate of this stage was relatively higher due to the numerous active sites on the surface of the adsorbent at the beginning of the adsorption process. In the second stage, the Cu^2+^ ions penetrated into the inner surface of the SA/PAA and 1.5 wt.%CNTs@SA/PAA adsorbents, in which the rates (k*_id_*_2_ = 1.664, 2.437) were lower than those of the first stage (k*_id_*_1_ = 23.671, 23.935). This might be caused by the adsorption process of heavy metal ions being controlled by pore diffusions [76]. In the third stage, the adsorption tended to equilibrate as the internal diffusion resistance increased with the gradual increase in copper ions inside the adsorbent [77]. 

#### 2.2.7. Adsorption Isotherm Analysis

The adsorption isotherm could be employed to elucidate the mechanism of interaction between the adsorbate and the adsorbent and analyze the energy characteristics of the adsorbent surface. The Langmuir, Freundlich, Temkin, Dubinin–Radushkevich (D–R), Sips, Toth, and Khan isotherm models were used to fit the adsorption experimental results to explain the adsorption mechanism of Cu^2+^ ions with the SA/PAA and CNTs@SA/PAA hydrogels.

**Figure 9:**
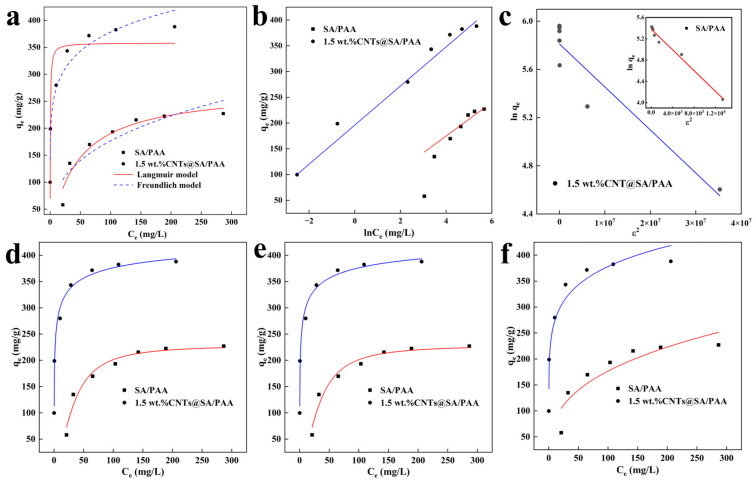
Adsorption isotherm of Cu^2+^ ions on the resultant SA/PAA and 1.5 wt.%CNTs@SA/PAA hydrogels. (**a**) Langmuir and Freundlich, (**b**) Temkin, (**c**) Dubinin–Radushkevich, (**d**) Sips, (**e**) Toth, (**f**) Khan. Adsorption conditions: 1.5 wt.%CNTs@SA/PAA hydrogel, adsorbent dosage 500 mg/L, pH 5, and temperature 25 °C.

The adsorption isotherm parameters were calculated using the models mentioned in Section 3.7.2, and the results are summarized in Figure 9 and Table 4, Table 5, Table 6 and Table 7. In Figure 9 and Table 4, the correlation coefficient, *R*^2^, shows that for the SA/PAA adsorbent, the best fitting is the Langmuir adsorption isotherm model, with an *R*^2^ of 0.92 and a saturated adsorption capacity of 273.71 mg/g, suggesting that the adsorption process was mainly a mono-molecular layer adsorption process. As for the 1.5 wt.%CNTs@SA/PAA adsorbent, the Freundlich adsorption isotherm model should be the model to describe the adsorption mechanism, suggesting that the adsorption process was mainly a multimolecular layer adsorption process, with an *R*^2^ of 0.93 and a saturated adsorption amount of 358.32 mg/g. As shown in Table 4, the *R_L_* values in the Langmuir adsorption isotherm model were in the range of 0 to 1, which suggests that the adsorption reactions of Cu^2+^ ions on the SA/PAA and 1.5 wt.%CNTs@SA/PAA adsorbents are favorable and spontaneous. *n* > 1 in the Freundlich adsorption isotherm model, which suggests that there are other adsorption factors influencing the adsorption process, such as ion exchange, electrostatic attraction, etc. [78]. 

There is not much difference in the *R*^2^ values of the two adsorbents, which were based on the Langmuir adsorption isothermal model. This suggests that the adsorption of Cu^2+^ ions with the resultant hydrogels combines physical and chemical interactions.

The Temkin isothermal adsorption line model reveals the occurrence of the interactions between the adsorbent and the adsorbate. This model is based on the following assumptions: the binding energy is uniformly distributed, the binding energy is maximum, and the heat of adsorption decreases linearly with surface coverage. The linearized form of the Temkin isotherm was obtained by plotting *q_e_* versus *lnC_e_* (Figure 9b), and the relevant simulation parameters are listed in Table 5.

The values of *A_T_* and *b* could be obtained from the slope and intercept of the line plot. The adsorption is an exothermic process due to the positive value of *b*. 

The *A_T_* value of the 1.5 wt.%CNTs@SA/PAA hydrogel was much higher than that of the SA/PAA hydrogel, suggesting that the affinity of this adsorbent for Cu^2+^ ions is stronger than that of SA/PAA. Furthermore, Temkin had the highest fitted isotherm *R*^2^ value (*R*^2^ = 0.99) relative to Langmuir and Freundlich adsorption isotherm models, revealing that there was a strong interaction between the Cu^2+^ ions and the 1.5 wt.%CNTs@SA/PAA hydrogels. The adsorption characteristics could be elucidated with a mixed monolayer–multilayer mechanism [79].

The Dubinin–Radushkevich isotherm was used to determine whether the adsorption mechanism is chemical or physical with free energy surfaces, and typically describes multilayer adsorption processes with van der Waals interaction forces. Figure 9c shows the linear relationship between ln *q_e_* and *ε*^2^. The theoretical adsorption parameters, *K_ad_* and *q_e_,* were calculated from the intercept and slope, respectively. To predict the possible adsorption mechanism of the model, the free energy, *E*, was determined according to Equation (12). The adsorption is physical adsorption, as *E* is less than 8 kJ/mol; ion-exchange adsorption, as the *E* is in the range of 8 to 16 kJ/mol; and chemical adsorption, as *E* is higher than 16 kJ/mol. As known from Table 6, the *E* values were less than 8 kJ/mol, indicating that the adsorption of Cu^2+^ ions was physisorption on both composite hydrogels [80].

Interestingly, compared to the Langmuir and Freundlich isotherms, the adsorption data of the SA/PAA adsorbent showed a better fit (*R*^2^ > 0.98). The maximum value of *q_m_* obtained from the linear regression was 217 mg/g, close to the experimental value of *q_m_*, 227 mg/g, suggesting the multilayer mode of physical adsorption of Cu^2+^ ions on its surface.

To further explore the adsorption mechanism of Cu^2+^ ions on the two adsorbents, the three-parameter nonlinear Sips, Toth, and Khan adsorption isotherm models were used for fitting. The results fitted with the experimental data are shown in Figure 9d–f and Table 7. The Sips isotherm is a combination of Langmuir and Freundlich expressions derived from the prediction of heterogeneous adsorption systems. It could be transformed into the Langmuir isotherm or Freundlich isotherm [81].

The exponents of the two adsorbents, *m* values, are shown in Table 7. Considering the correlation coefficient (*R*^2^) of 1.5 wt.%CNTs@SA/PAA in the two-parameter Langmuir and Freundlich models, it could be inferred that the adsorption data of 1.5 wt.%CNTs@SA/PAA are more biased towards the Freundlich form of multilayer adsorption rather than the Langmuir form. The Toth isotherm model has been used to describe the adsorption in heterogeneous systems, based on the assumption that the adsorption energy at most sites is less than the mean value. As the model exponent, *m*, is equal to 1, the isotherm reduces to the Langmuir adsorption isotherm equation. As known from Table 4, the exponents, 1/*m*, for the two adsorbents, do not approach 1 with Toth isotherm models. The *a_k_,* devoted to the model exponent in the Khan isotherm model distance, is quite different, and the same results are obtained with the Sips isotherm model and the Toth isotherm model, which are more inclined to the Freundlich model. Therefore, the heavy metal Cu^2+^ ions in the two adsorbent composites might be assumed to be through a multilayer adsorption mechanism.

### 2.3. Thermodynamics Analysis

Table 8 shows the equilibrium constants and Gibbs free energy (ΔG°) of adsorption of Cu^2+^ ions on the SA/PAA and 1.5 wt.%CNTs@SA/PAA composite hydrogels. In the Cu^2+^ adsorption system, the ΔG° of the SA/PAA and 1.5 wt.%CNTs@SA/PAA composite hydrogels were −8.92 and −23.36 kJ/mol, respectively, both of which were negative, suggesting the spontaneity of the adsorption of Cu^2+^ ions. The absolute value of ΔG° increased due to the doping of the CNTs, further proving a more favorable adsorption state by employing the 1.5 wt.%CNTs@SA/PAA composite hydrogel as the adsorbent. 

### 2.4. Mechanism 

**Figure 10:**
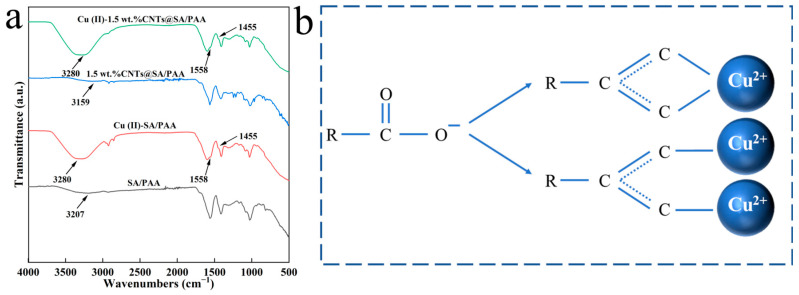
(**a**) FTIR spectra of SA/PAA and 1.5 wt.%CNTs@SA/PAA composite hydrogels before and after the adsorption of Cu^2+^ ions. (**b**) The possible adsorption mechanism of Cu^2+^ ions with 1.5 wt.%CNTs@SA/PAA hydrogel.

Through the discussion of adsorption kinetics, adsorption isotherms, and adsorption thermodynamics, it could be found that adsorption is the combination of the interaction of multiple mechanisms, such as the electrostatic interaction, the ion exchange, the chelation, the complexation, etc. The adsorption mechanism of the 1.5 wt.%CNTs@SA/PAA hydrogel was further analyzed using FTIR spectroscopy. Figure 10 shows the related FTIR spectra. As clearly seen, the peak of –OH of the SA/PAA hydrogel shifted from 3207 cm^−1^ for to 3280 cm^−1^ due to the binding of Cu^2+^ and –OH groups, while that of 1.5 wt.%CNTs@SA/PAA shifted from 3159 cm^−1^ to 3273 cm^−1^, suggesting that the hydroxyl groups and Cu^2+^ ions were involved in adsorption and formation of the complexes [82]. The new absorption peaks at 1558 cm^−1^ and 1455 cm^−1^ that appeared after the adsorption of Cu^2+^ ions with the two adsorbents could be assigned to the asymmetric stretching vibration (*ν_asym_*) and symmetric stretching vibration (*ν_sym_*) of the carboxylate. The degree of separation Δν (*ν*_*asym*−_*ν*_*sym*_) of carboxylate groups is commonly used to characterize the interaction form of carboxylate groups. Δν, herein 103 cm^−1^, is much less than Δν_Na_ reported in the literature (Figure 10b), indicating that the carboxylic acid complexes are bidentate coordination containing bidentate chelation and bidentate bridging, which is more favorable for the adsorption of Cu^2+^ ions [82,83]. 

It could be found from Figure 10a that there is nearly no change in the FTIR spectrum of the 1.5 wt.%CNTs@SA/PAA hydrogels before and after the adsorption of Cu^2+^ ions. After Cu^2+^ adsorption, the intensities of the characteristic peaks increased, suggesting chemical bonding between the –COOH, –OH, and Cu^2+^ ions.

**Figure 11:**
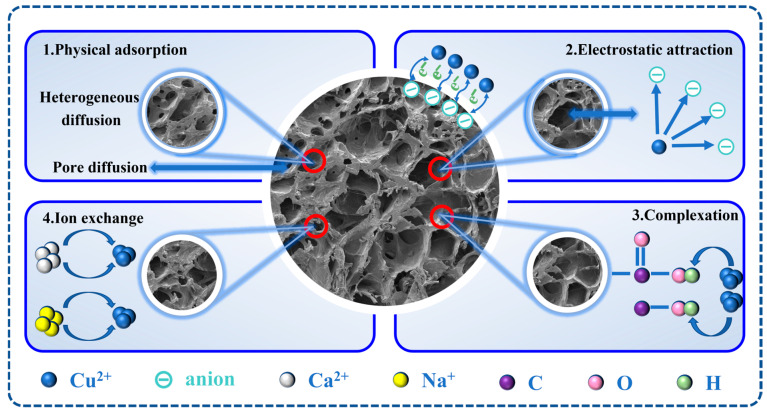
Schematic diagram of the mechanism of Cu^2+^ ion removal with CNTs@SA/PAA composite hydrogel.

Figure 11 provides a possible mechanism for the adsorption of Cu^2+^ with the CNTs@SA/PAA hydrogels. Both the electrostatic interaction between the Cu^2+^ ions and carboxyl groups and the ion exchange of a Cu^2+^ ion with Na^+^ are favorable for removing Cu^2+^ from water and wastewater. Additionally, complexation occurred, as Cu^2+^ ions form ionic bonds with two carboxyl groups simultaneously, and non-homogeneous diffusion triggered by the porous structure of the CNTs@SA/PAA hydrogel facilitates the removal of Cu^2+^ ions.

## 3. Materials and Methods

### 3.1. Chemicals and Reagents

SA, AA, CNT, and calcium chloride anhydrous (CaCl_2_) were obtained from Macklin Chemical Reagent Company (Shanghai, China). MBA and sodium dodecyl sulfate (SDS) were purchased from Sinopharm Chemical Reagents Co., Ltd. (Shanghai, China). *N*,*N*,*N*,*N*-Tetramethylethylenediamine (TEMED), and copper sulfate pentahydrate (CuSO_4_·5H_2_O) were supplied by RON reagent Co., Ltd. (Shanghai, China). Sodium hydroxide (NaOH) and APS were purchased from Aladdin Co., Ltd. (Shanghai, China). All the chemical reagents were of analytical grade and used directly without further purification. Ultrapure water (UPW) was used in this study. 

### 3.2. Preparation of SA/PAA and CNTs@SA/PAA Hydrogel

The CNT-doped SA/PAA composite hydrogel was prepared with free radical polymerization. Approximately 10 mg CNT and 1 mL SDS were dispersed in 20 mL UPW, then ultrasonicated (250 w, 40 KHz) for 30 min. The solution was poured into a 100 mL three-necked flask. After the additions of 1 g AA and 0.44 g NaOH, the CNTs solution was put in an oil bath at 68 °C for 10 min while purged with nitrogen. After 30 mg MBA was added to the solution, 10 mg APS was added to the mixture to produce free radicals under stirring at 200 rpm for 30 min. Then, 20 µL TEMED and 1 g SA were dispersed slowly into the solution. The mixture was then continuously stirred at 200 rpm for 5 h at room temperature. The solution was gradually dropped into a 400 mL 4% (*w*/*v*) CaCl_2_ aqueous solution, then set for 24 h for hardening. These composites were named 0.5 wt.%CNTs@SA/PAA, 1.5 wt.%CNTs@SA/PAA, and 2.5 wt.%CNTs@SA/PAA, according to the weight concentration of the CNTs. The preparation process of the SA/PAA hydrogel was the same as that of the CNTs@SA/PAA.

### 3.3. Characterization of Resultant Hydrogels

The 2-D morphologies were observed with a cold-field emission scanning electron microscope (S-4800, Hitachi, Tokyo, Japan). The chemical structures were characterized with an FT-IR spectrometer (Spectrum Two, PerkinElmer, Waltham, MA, USA). The X-ray diffraction patterns were characterized with an X-ray diffractometer (Smartlab 9 kW, Rigaku Corporation, Akishima, Japan). The structures were visualized with a transmission electron microscope (FEI Tecnai G2 F20 S-TWIN, FEI Company, Hillsboro, OR, USA). 

### 3.4. Determination of Cu^2+^ Concentrations 

The Cu^2+^ concentrations were determined with an atomic absorption spectrophotometer (AAS, TAS-990, Beijing Purkinje General Instrument Co., Ltd., Beijing, China).

### 3.5. Swelling Equilibria

The resultant hydrogels weighing 300 mg were soaked in 1000 mL UPW for 24 h. Then, the weights of the swollen hydrogels were measured. The swelling rate (%) could be calculated with Equation (1).

(1) $$SR\left(\%\right)=\frac{W_{t}-W_{d}}{W_{d}}$$

where *W_t_* and *W_d_* are the weights of the swollen and dry hydrogels, respectively.

### 3.6. Batch Adsorption Experiments

The measurements of the Cu^2+^ adsorbed were carried out with batch adsorption experiments, and the effect of the CNTs’ content doped on the adsorption capacity of the resultant hydrogel was investigated. CuSO_4_·5H_2_O was selected as the source of Cu^2+^ ions. All the adsorption experiments were carried out at a speed of 200 rpm in a rotary oscillator with a 50 mL falcon tube at room temperature. The effect of the pH in the range of 1–6 on the adsorption capacity of the hydrogel was investigated. The adsorption kinetics were investigated under the following conditions: initial Cu^2+^ concentration of 100 mg/L, adsorption time range of 0 to 360 min. The adsorption isotherms were investigated under the following conditions: Cu^2+^ concentration in the range of 50 to 400 mg/L and an adsorption time of 24 h. According to the difference of ion concentrations in the solution before and after adsorption, the removal efficiency (*R_e_*) and equilibrium adsorption (*q_e_*) capacity of Cu^2+^ were calculated as Equations (2) and (3).

(2) $$R_{e}\left(\%\right)=\frac{\left(C_{0}-C_{e}\right)\;}{C_{0}}\times 100$$

where *C*_0_ is the initial adsorbate concentration (mg/L) and *C_e_* is the adsorbate concentration (mg/L) at the liquid-phase equilibrium.

(3) $$q_{e}=\frac{\left(C_{0}-C_{e}\right)\times V}{M}$$

where *C*_0_ (mg/L) and *C_e_* (mg/L) are the initial and equilibrium concentrations of Cu^2+^, respectively; *V* represents the volume of adsorbate (L); and *M* is the weight of the adsorbent (g).

### 3.7. Mathematical Modeling

#### 3.7.1. Adsorption Kinetics

Two kinetic models frequently used in the adsorption behaviors of hydrogels, the pseudo-first order (PFO) and pseudo-second order (PSO), were employed to evaluate the adsorption characteristics of the resultant CNTs@SA/PAA composite hydrogels. The linear kinetic models are represented by Equations (4) and (5), respectively.

Pseudo-first order:

(4) $$ln\left(q_{e}-q_{t}\right)=\ln q_{e}-\frac{k_{1}}{2.303}t$$

Pseudo-second order:

(5) $$\frac{t}{q_{t}}=\frac{1}{k_{2}\;q_{e}^{2}}+\frac{t}{q_{e}}$$

where *k*_1_ (1/min) and *k*_2_ (g/(mg·min)) are the rate constant of the pseudo-first order and pseudo-second order of adsorptions, respectively. *q_t_* (mg/g) is the adsorption capacity at time *t* and *q_e_* (mg/g) is the adsorption capacity at equilibrium and time *t*.

The kinetics of the chemical adsorption mechanism were also investigated using the Elovich model, expressed as the following equation [84]:

(6) $$q_{t}=\frac{1}{\beta }\;\ln\;\left(\alpha \beta t+1\right)$$

where *α* (mg/(g·min)) is the initial adsorption rate and *β* (g/mg) is the desorption constant relating to the surface coverage and the chemical adsorption activation energy. 

The intraparticle diffusion model was employed to conduct further investigation, as this model could identify the reaction pathways, predicting the dominant or controlling steps of the adsorption rate [85].

(7) $$q_{t}=k_{id}t^{1/2}+C$$

where *q_t_* is the adsorption capacity of Cu^2+^ ions (mg/g) at time *t*, *k_id_* is the intraparticle diffusion coefficient (mg/(g·min^0.5^)), and *C* is the boundary layer effect (mg/g). The value of *C* is proportional to the thickness of the boundary layer; the larger the *C* value, the greater the effect of the thickness of the diffusion boundary layer.

#### 3.7.2. Adsorption Isotherms

The adsorption isotherm illustrates the adsorption equilibrium, indicating the proportion of the adsorption capacity to the equilibrium concentration or the pressure at a constant temperature. The isothermal adsorption line presents the equilibrium state of the adsorbate molecules between the solid and liquid phases [86]. 

The adsorption isotherm could qualitatively analyze and determine the adsorption type and explain the interaction between the adsorbate and the surface selective sites of the adsorbent. The adsorption characteristics of the SA/PAA and CNTs@SA/PAA hydrogels for Cu^2+^ ions could be expressed by the adsorption isotherm, which plays an important role in inferring the adsorption mechanism and evaluating the adsorption capacity of adsorbents.

In this study, the Langmuir, Freundlich, Temkin, Dubinin–Radushkevich (D–R), Sips, Toth, and Khan isotherm models were employed to fit the experimental data and analyze the adsorption behaviors of the Cu^2+^ by SA/PAA and CNTs@SA/PAA hydrogels. The derivation and establishment of the Langmuir isotherm model are based on the following three assumptions: (I) a single molecule layer formed on the surface of the adsorbent; (II) the interaction between the molecules adsorbed at adjacent positions could be ignored; (III) all the adsorption sites are the same and have equivalent energy, and the possibility of utilizing adsorption sites is the same. This model could be expressed with the following Equation (8) [87].

(8) $$\mathrm{Langmuir}:\;q_{e}=\frac{q_{m}K_{L}C_{e}}{1+K_{L}C_{e}}$$

where *C_e_* is the equilibrium concentration of the Cu^2+^ solution (mg/L), *q_e_* is the equilibrium adsorption capacity (mg/g), *q_m_* (mg/g) is the maximum monolayer adsorption capacity, and *K_L_* (L/mg) is the Langmuir equilibrium constant. The intercept and slope values of the curve are used to determine the *K_L_* and *q_m_* values, respectively.

The Langmuir isotherm reveals an important equilibrium parameter known as the separation factor (*R_L_*). The *R_L_* could be calculated with the Equation (9) [88].

(9) $$R_{L}=\frac{1}{1+K_{L}C_{0}}$$

where *C_0_* (mg/L) is the initial concentration of the Cu^2+^ ions. *R_L_* is used to describe the experimental efficiency and the shape. The adsorption is linearly irreversible, as *R_L_* is 1 and 0, respectively. The adsorption is unfavorable when *R_L_* > 1, while it is favorable when *R_L_* is in the range of 0 to 1 [89]. 

Different from the Langmuir model, the Freundlich isotherm is based on the multilayer adsorption of metal ions on the heterogeneous surface of adsorbents, where the adsorption sites are not equivalent and the adsorption heat is uneven. This isotherm could be expressed with Equation (10) [90].

(10) $$\mathrm{Freundlich}:\;q_{e}=K_{F}C_{e}^{1/n}$$

where *q_e_* is the equilibrium adsorption capacity (mg/g), *C_e_* (mg/L) is the equilibrium concentration of the Cu^2+^ ions (mg/L), *K_F_* is the empirical constant of the Freundlich isotherm, and *n* is the empirical parameter related to the favorability of the adsorption process. The intercept and the slope value of *q_e_* versus ln *C_e_* plot were used to calculate the *K_F_* and *n* values, respectively. 

The Temkin isotherm was also used to calculate the adsorption heat change arising from the interaction between the adsorbent and the adsorbed substance. The Temkin equation is presented with Equation (11) [91].

(11) $$q_{e}=\frac{RT}{b}\ln A_{T}+\frac{RT}{b}\ln C_{e}$$

where *A_T_* (L/g) is the Temkin isotherm binding constant, *b* is the Temkin heat of adsorption coefficient (J/mol), *R* is the universal gas parameter (8.314 J/mol/K), and *T* is the absolute temperature (K), respectively.

The mathematical equivalence of the Dubinin–Radushkevich (D–R) isotherms provides additional information regarding the adsorption mechanism on heterogeneous surfaces with Equations (12)–(14) [92].

(12) $$\ln q_{e}=\ln q_{m}-K_{ad}\;(\varepsilon^{2})$$

(13) $$\varepsilon =RT\ln\left[1+\frac{1}{C_{e}}\right]$$

(14) $$E=\frac{1}{\sqrt{2K}}$$

where *q_m_* (mg/g) is the adsorption capacity, *K_ad_* is the energy of adsorption constant (mol^2^/kJ^2^), ε is the Dubinin–Radushkevich isotherm constant, *R* is, and the universal gas parameter (8.314 J/mol/K), and *T* is the absolute temperature (K), respectively. The average energy of the adsorption process (*E*) could predict the adsorption mechanism.

The Sips isotherm is expressed by Equation (15) [93].

(15) $$q_{e}=\frac{q_{s}{\left(K_{s}C_{e}\right)}^{m}}{1+{\left(K_{s}C_{e}\right)}^{m}}$$

where *q_s_* is the adsorption capacity of the adsorbent, *K_S_* is the equilibrium constant (1/mg), and *m* is the Sips isotherm equation exponent. If *m* converges to 1 or is equal to 1, the model tends towards the Langmuir equation; otherwise, if the *C_e_* or *K_s_* approach 0, the well-known Freundlich isotherm equation is reduced. 

The Toth model is expressed by Equation (16) [94].

(16) $$q_{e}=\frac{q_{m}C_{e}}{{\left(a_{T}+C_{e}\right)}^{1/m}}$$

where *q_e_* is the adsorbed amount at equilibrium (mg/g), *C_e_* is the equilibrium concentration of the absorbate (mg/L), *q_m_* is the maximum adsorption capacity according to the Toth model (mg/g), *a_T_* is the Toth equilibrium constant, and *m* is the exponent in the Toth model equation. If the value of 1/*m* approaches 1, it is advisable to employ the Langmuir model. Otherwise, the Freundlich model is deemed more suitable.

The Khan model is expressed by Equation (17) [94].

(17) $$q_{e}=\frac{q_{s}b_{K}C_{e}}{{\left(1+C_{e}b_{K}\right)}^{a_{K}}}$$

Here, *a_K_* serves as the model exponent and *b_K_* is a model constant. If *a_K_* approaches 1, the adsorption aligns well with the Langmuir model; otherwise, the Freundlich model is deemed to be the most appropriate.

### 3.8. Adsorption Thermodynamics

The Gibbs free energy could be calculated using the thermodynamic equilibrium constant *K*_0_, which is defined as the following Equation (18) [81].

(18) $$K_{0}=\frac{a_{s}}{a_{e}}=\frac{v_{s}q_{e}}{v_{e}C_{e}}$$

where *a_s_* is the activity of adsorbed Cu^2+^, *a_e_* is the activity of Cu^2+^ in the equilibrium state, *ν_s_* is the activity coefficient of adsorbed Cu^2+^, and *ν_e_* is the activity coefficient of Cu^2+^ in the equilibrium state. 

As the Cu^2+^ concentration in the solution decreases and approaches zero, the activity coefficient *ν* is close to 1. Equation (18) could be written as the following Equation (19).

(19) $$\underset{q_{e}\to 0}{\lim}=\frac{a_{s}}{a_{e}}=\frac{q_{e}}{C_{e}}=K_{0}$$

*K_0_* could be obtained by plotting ln (*q_e_/C_e_*) versus *q_e_* and extrapolating *q_e_* to zero. Its intercept gives the value of *K*_0_.

The change of the standard free energy of adsorption, ΔG° (kJ/mol), could be calculated according to the following Equation (20).

(20) $$\Delta G{}^{\circ}=-RT\ln K_{0}$$

where *R* is the general gas constant of 8.314 × 10^−3^ kJ/K/mol and *T* is the absolute temperature (K).

### 3.9. Statistical Analysis

The data were analyzed as the mean ± standard deviation (*SD*) of three determinations. 

## 4. Conclusions

This study presents the preparation of green hydrogels, specifically, SA/PAA and CNTs@SA/PAA composite hydrogels, via free radical polymerization. The adsorption capabilities of the resultant hydrogels for Cu^2+^ ions were investigated under different conditions. The characterizations revealed that both the SA/PAA and CNTs@SA/PAA composite hydrogels have 3-D network structures with numerous pores, which could be the adsorption sites for Cu^2+^ ions. The FTIR spectra suggested the involvement of –OH and –COOH groups in the adsorption process. The rheological and EDS analyses demonstrated that the doping of CNTs enhanced the mechanical properties and stability of the resultant composite hydrogels. The DSC revealed a 22% increase in the *T_g_* value of the resultant CNTs@SA/PAA composite hydrogel. The optimal adsorption capacity of the CNTs@SA/PAA hydrogel was achieved at 1.5 wt.%CNTs, pH 5.0, and a dosage of 500 mg/L. The adsorption equilibrium of Cu^2+^ ions was reached after 360 min. The pseudo-second order kinetic model and the Freundlich isotherm model showed good fits with the adsorption kinetic data and the equilibrium adsorption isotherms. According to the Freundlich model, the maximum adsorption capacity at room temperature was 358.32 mg/g. The dimensionless equilibrium parameter, *R_L_*, suggested that the adsorption is favorable and spontaneous, further supported by the thermodynamic analysis. It could be concluded that the resultant CNTs@SA/PAA hydrogel is an ideal bio-adsorbent for the removal of Cu^2+^ ions from water and wastewater due to its high efficiency, low cost, and stability. Although the positive results herein are acknowledged, multiple heavy metal ion absorption experiments are required to be undertaken in the near future. 

## Figures and Tables

**Table 1:** Kinetic parameters of the pseudo-first order and pseudo-second order kinetic models for Cu^2+^ adsorption of the resultant hydrogels.

Adsorbent	*q_e_*	Pseudo-First Order Kinetic Model			Pseudo-Second Order Kinetic Model		
		*K*_1_ × 10^−3^	*q*_1*e*_	*R* ^2^	*K*_2_ × 10^−3^	*q*_2*e*_	*R* ^2^
SA/PAA	171.570	14.1	44.057	0.8852	1.045	173.913	0.9999
1.5 wt.%CNTs@SA/PAA	191.570	14.3	63.194	0.9445	4.595	194.553	0.9998

**Table 2:** Parameters and correlation coefficients were obtained from the analysis of adsorption kinetics with the Elovich equation.

Adsorbent	Elovich		
	*α*	*β*	*R* ^2^
SA/PAA	100.797	0.011	0.906
1.5 wt.%CNTs@SA/PAA	109.581	0.010	0.948

**Table 3:** Parameters and correlation coefficients based on the analysis of adsorption kinetics with the intraparticle diffusion model.

Adsorbent	Intraparticle Diffusion								
	First Stage			Second Stage			Third Stage		
	*k_id_*_1_	*C*	*R* ^2^	*k_id_*_2_	*C*	*R* ^2^	*k_id_*_3_	*C*	*R* ^2^
SA/PAA	23.671	27.792	0.971	1.664	146.497	0.962	0.534	161.452	0.9996
1.5 wt.%CNTs@SA/PAA	23.935	38.842	0.968	2.437	153.192	0.982	1.125	170.648	0.944

**Table 4:** The calculated parameters for Langmuir and Freundlich isotherm models of SA/PAA and 1.5 wt.%CNTs@SA/PAA.

Analyte	Langmuir Model				Freundlich Model		
	*q_m_*	*K_L_*	*R_L_*	*R* ^2^	*n*	*K_F_*	*R* ^2^
SA/PAA	273.71	0.02	0.111–0.500	0.92	2.98	37.74	0.80
1.5 wt.%CNTs@SA/PAA	358.32	2.41	0.001–0.008	0.88	7.07	197.168	0.93

**Table 5:** The parameters for Temkin isotherm models of the resultant SA/PAA and 1.5 wt.%CNTs@SA/PAA hydrogels.

Analyte	*A_T_*	*b* (J/mol)	*R* ^2^
SA/PAA	3.89333	75.8828	0.82
1.5 wt.%CNTs@SA/PAA	174.868	65.2336	0.99

**Table 6:** Dubinin–Radushkevich (D–R) constants for Cu^2+^ ions using SA/PAA and 1.5 wt.%CNTs@SA/PAA hydrogels as the adsorbents.

Analyte	*K* (mol^2^/J^2^)	*q_m_* (mg/g)	*E* (kJ/mol)	*R* ^2^
SA/PAA	9.78 × 10^−5^	217.022	0.07	0.98
1.5 wt.%CNTs@SA/PAA	3.57 × 10^−8^	333.619	3.74	0.86

**Table 7:** Three parameter isotherm factors for Cu^2+^ ion adsorption onto the SA/PAA and 1.5 wt.%CNTs@SA/PAA composite hydrogels.

Isotherms	Parameters	SA/PAA	1.5 wt.%CNTs@SA/PAA
Sips	*K_s_*	0.03	0.52
	*q_s_*	228.89	460.15
	*m*	1.76	0.38
	*R* ^2^	0.95	0.98
Results		Freundlich	Freundlich
Toth	*q_m_*	37.77	197.29
	*a_T_*	3.86	1.00
	*m*	1.50	1.16
	*R* ^2^	0.74	0.91
Results		Freundlich	Freundlich
Khan	*q_s_*	538.23	194.79
	*b_K_*	0.009	9.38
	*a_K_*	1.42	0.90
	*R* ^2^	0.92	0.98
Results		Freundlich	Freundlich

**Table 8:** Equilibrium constant and Gibbs free energy of adsorption of Cu^2+^ on SA/PAA and 1.5 wt.%CNTs@SA/PAA.

	SA/PAA	1.5 wt.%CNTs@SA/PAA
Intercept, lnK_0_	3.60	9.43
ΔG° (kJ/mol)	−8.92	−23.36
R^2^	0.91	0.96

## Data Availability

Data are contained within the article.

## References

[1] Chen Y.-G., He X.-L.-S., Huang J.-H., Luo R., Ge H.-Z., Wołowicz A., Wawrzkiewicz M., Gładysz-Płaska A., Li B., Yu Q.-X. (2021). Impacts of heavy metals and medicinal crops on ecological systems, environmental pollution, cultivation, and production processes in China. Ecotox. Environ. Safe.

[2] Sener S., Sener E., Varol S. (2020). Hydro-chemical and microbiological pollution assessment of irrigation water in Kizilirmak Delta (Turkey). Environ. Pollut..

[3] Tesseme A.T., Vinti G., Vaccari M. (2022). Pollution potential of dumping sites on surface water quality in Ethiopia using leachate and comprehensive pollution indices. Environ. Monit. Assess..

[4] Rather R.A., Ara S., Padder S.A., Sharma S., Pathak S.P., Baba T.R. (2023). Seasonal fluctuation of water quality and ecogenomic phylogeny of novel potential microbial pollution indicators of Veshaw River Kashmir-Western Himalaya. Environ. Pollut..

[5] Ren W., Wu X., Yang J., Luo L., Liang S., Yang H. (2022). Water pollution characteristics of inflowing rivers under different land-use patterns in the Daye Lake basin: Pollution mode and management suggestions. Environ. Monit. Assess..

[6] Godiya C.B., Sayed S.M., Xiao Y., Lu X. (2020). Highly porous egg white/polyethyleneimine hydrogel for rapid removal of heavy metal ions and catalysis in wastewater. React. Funct. Polym..

[7] Pooja G., Kumar P.S., Indraganti S. (2022). Recent advancements in the removal/recovery of toxic metals from aquatic system using flotation techniques. Chemosphere.

[8] Ma J., Luo J., Liu Y., Wei Y., Cai T., Yu X., Liu H., Liu C., Crittenden J.C. (2018). Pb(II), Cu(II) and Cd(II) removal using a humic substance-based double network hydrogel in individual and multicomponent systems. J. Mater. Chem. A.

[9] Mahapatra M., Karmakar M., Dutta A., Mondal H., Roy J.S.D., Chattopadhyay P.K., Singh N.R. (2018). Microstructural analyses of loaded and/or unloaded semisynthetic porous material for understanding of superadsorption and optimization by response surface methodology. J. Environ. Chem. Eng..

[10] Zhang L., Wang J., Ren X., Zhang W., Zhang T., Liu X., Du T., Li T., Wang J. (2018). Internally extended growth of core–shell NH2-MIL-101(Al)@ZIF-8 nanoflowers for the simultaneous detection and removal of Cu(II). J. Mater. Chem. A.

[11] Zhang X., Huang P., Zhu S., Hua M., Pan B. (2019). Nanoconfined Hydrated Zirconium Oxide for Selective Removal of Cu(II)-Carboxyl Complexes from High-Salinity Water via Ternary Complex Formation. Environ. Sci. Technol..

[12] Sun Y., Yang X., Zhang R., Xia T., Hu K., Hao F., Liu Y., Deng Q., Yang S., Wen X. (2023). One-step effervescence tablet-assisted switchable hydrophilic solvent microextraction combined with micro spectrophotometry for the determination of copper in Salvia yunnanensis and environmental samples. Microchem. J..

[13] Saravanan A., Kumar P.S., Ramesh B., Srinivasan S. (2022). Removal of toxic heavy metals using genetically engineered microbes: Molecular tools, risk assessment and management strategies. Chemosphere.

[14] WHO (2022). Guidelines for Drinking-Water Quality: Fourth Edition Incorporating the First and Second Addenda. https://www.who.int/publications/i/item/9789240045064.

[15] Shao J., Shao P., Peng M., Li M., Yao Z., Xiong X., Qiu C., Zheng Y., Yang L., Luo X. (2023). A pyrazine based metal-organic framework for selective removal of copper from strongly acidic solutions. Front. Environ. Sci. Eng..

[16] Bandara T., Xu J., Potter I.D., Franks A., Chathurika J.B.A.J., Tang C. (2020). Mechanisms for the removal of Cd (II) and Cu (II) from aqueous solution and mine water by biochars derived from agricultural wastes. Chemosphere.

[17] Li J., Zheng L., Wang S.-L., Wu Z., Wu W., Niazi N.K., Shaheen S.M., Rinklebe J., Bolan N., Ok Y.S. (2019). Sorption mechanisms of lead on silicon-rich biochar in aqueous solution: Spectroscopic investigation. Sci. Total Environ..

[18] Li M., Meng X., Huang K., Feng J., Jiang S. (2019). A novel composite adsorbent for the separation and recovery of indium from aqueous solutions. Hydrometallurgy.

[19] Taylor A.A., Tsuji J.S., Garry M.R., McArdle M.E., Goodfellow W.L., Adams W.J., Menzie C.A. (2020). Critical Review of Exposure and Effects: Implications for Setting Regulatory Health Criteria for Ingested Copper. Environ. Manag..

[20] Krstic V., Urosevic T., Pesovski B. (2018). A review on adsorbents for treatment of water and wastewaters containing copper ions. Chem. Eng. Sci..

[21] Shi X.-L., Chen Y., Hu Q., Zhang W., Luo C., Duan P. (2017). A potential industrialized fiber-supported copper catalyst for one-pot multicomponent CuAAC reactions in water. J. Ind. Eng. Chem..

[22] Barrera-Diaz C.E., Lugo-Lugo V., Bilyeu B. (2012). A review of chemical, electrochemical and biological methods for aqueous Cr(VI) reduction. J. Hazard. Mater..

[23] Zhang L., Zhao Y.-H., Bai R. (2011). Development of a multifunctional membrane for chromatic warning and enhanced adsorptive removal of heavy metal ions: Application to cadmium. J. Membr. Sci..

[24] Qi X., Liu R., Chen M., Li Z., Qin T., Qian Y., Zhao S., Liu M., Zeng Q., Shen J. (2019). Removal of copper ions from water using polysaccharide-constructed hydrogels. Carbohydr. Polym..

[25] Karim M.R., Aijaz M.O., Alharth N.H., Alharbi H.F., Al-Mubaddel F.S., Awual M.R. (2019). Composite nanofibers membranes of poly(vinyl alcohol)/chitosan for selective lead(II) and cadmium(II) ions removal from wastewater. Ecotox. Environ. Safe.

[26] Liu M., Jia L., Zhao Z., Han Y., Li Y., Peng Q., Zhang Q. (2020). Fast and robust lead (II) removal from water by bioinspired amyloid lysozyme fibrils conjugated with polyethyleneimine (PEI). Chem. Eng. J..

[27] Wang G., Lu T., Zhang X., Feng M., Wang C., Yao W., Zhou S., Zhu Z., Ding W., He M. (2021). Structure and properties of cellulose/HAP nanocomposite hydrogels. Int. J. Biol. Macromol..

[28] Wong S.M., Zulkifli M.Z.A., Nordin D., Teow Y.H. (2021). Synthesis of Cellulose/Nano-hydroxyapatite Composite Hydrogel Absorbent for Removal of Heavy Metal Ions from Palm Oil Mill Effluents. J. Polym. Environ..

[29] Yang H.-R., Li S.-S., Shan X.-C., Yang C., An Q.-D., Zhai S.-R., Xiao Z.-Y. (2022). Hollow polyethyleneimine/carboxymethyl cellulose beads with abundant and accessible sorption sites for ultra-efficient chromium (VI) and phosphate removal. Sep. Purif. Technol..

[30] Kamcev J., Paul D.R., Freeman B.D. (2015). Ion Activity Coefficients in Ion Exchange Polymers: Applicability of Manning’s Counterion Condensation Theory. Macromolecules.

[31] Chen Q., Yao Y., Li X., Lu J., Zhou J., Huang Z. (2018). Comparison of heavy metal removals from aqueous solutions by chemical precipitation and characteristics of precipitates. J. Water. Process. Eng..

[32] Almomani F., Bhosale R., Khraisheh M., Kumar A., Almomani T. (2020). Heavy metal ions removal from industrial wastewater using magnetic nanoparticles (MNP). Appl. Surf. Sci..

[33] Pooja G., Kumar P.S., Prasannamedha G., Varjani S., Vo D.-V.N. (2021). Sustainable approach on removal of toxic metals from electroplating industrial wastewater using dissolved air flotation. J. Environ. Manag..

[34] Rahman M.A., Lamb D., Rahman M.M., Bahar M.M., Sanderson P., Abbasi S., Bari A.S.M.F., Naidu R. (2021). Removal of arsenate from contaminated waters by novel zirconium and zirconium-iron modified biochar. J. Hazard. Mater..

[35] Maapola P., Iraola-Arregui I., du Preez L., Okoro O.V., Görgens J.F. (2021). An Investigation into the Applicability of Pyrolyzed Tyre Char and Tyre Crumb for the Recovery of Gold from Acidic Solutions. Waste Biomass Valorization.

[36] Tan W.S., Ting A.S.Y. (2014). Alginate-immobilized bentonite clay: Adsorption efficacy and reusability for Cu(II) removal from aqueous solution. Bioresour. Technol..

[37] Huang D., Wang W., Wang A. (2013). Removal of Cu^2+^ and Zn^2+^ Ions from aqueous solution using sodium alginate and attapulgite composite hydrogels. Adsorpt. Sci. Technol..

[38] Ge Y., Cui X., Liao C., Li Z. (2017). Facile fabrication of green geopolymer/alginate hybrid spheres for efficient removal of Cu(II) in water: Batch and column studies. Chem. Eng. J..

[39] Wang Y., Feng Y., Zhang X.-F., Zhang X., Jiang J., Yao J. (2018). Alginate-based attapulgite foams as efficient and recyclable adsorbents for the removal of heavy metals. J. Colloid Interface Sci..

[40] Algothmi W.M., Bandaru N.M., Yu Y., Shapter J.G., Ellis A.V. (2013). Alginate-graphene oxide hybrid gel beads: An efficient copper adsorbent material. J. Colloid Interface Sci..

[41] Li J., Wang X., Zhao G., Chen C., Chai Z., Alsaedi A., Hayat T., Wang X. (2018). Metal-organic framework-based materials: Superior adsorbents for the capture of toxic and radioactive metal ions. Chem. Soc. Rev..

[42] Kimura M., Takai M., Ishihara K. (2007). Biocompatibility and drug release behavior of spontaneously formed phospholipid polymer hydrogels. J. Biomed. Mater. Res. Part A.

[43] Kong C., Zhao X., Li Y., Yang S., Chen Y.M., Yang Z. (2020). Ion-Induced Synthesis of Alginate Fibroid Hydrogel for Heavy Metal Ions Removal. Front. Chem..

[44] Tang Q., Sun X., Li Q., Wu J., Lin J. (2009). Synthesis of polyacrylate/polyethylene glycol interpenetrating network hydrogel and its sorption of heavy-metal ions. Sci. Technol. Adv. Mater..

[45] Akter M., Bhattacharjee M., Dhar A.K., Rahman F.B.A., Haque S., Rashid T.U., Kabir S.M.F. (2021). Cellulose-Based Hydrogels for Wastewater Treatment: A Concise Review. Gels.

[46] Godiya C.B., Cheng X., Li D., Chen Z., Lu X. (2019). Carboxymethyl cellulose/polyacrylamide composite hydrogel for cascaded treatment/reuse of heavy metal ions in wastewater. J. Hazard. Mater..

[47] Qian Y., Zhang J., Yu Y., Jiang Q., Yu X., Cheng Z. (2022). Preparation and release of novel frog egg-like microspheres by coaxial electrostatic spray technique. Mater. Lett..

[48] Wu Z., Han Y., Zan F., Ye Y., Ren Y., Han K., Liu D., Jiang W. (2023). Highly efficient removal of phosphate by La-diatomite and sodium alginate composite hydrogel beads. Environ. Sci.-Water Res..

[49] Gao X., Guo C., Hao J., Zhao Z., Long H., Li M. (2020). Adsorption of heavy metal ions by sodium alginate based adsorbent-a review and new perspectives. Int. J. Biol. Macromol..

[50] Zeng X., Zhang G., Wen J., Li X., Zhu J., Wu Z. (2023). Simultaneous removal of aqueous same ionic type heavy metals and dyes by a magnetic chitosan/polyethyleneimine embedded hydrophobic sodium alginate composite: Performance, interaction and mechanism. Chemosphere.

[51] Rahaman M.H., Islam M.R., Islam R., Alam S.M.N., Rahman M.S., Rahman M.A., Begum B.A. (2024). Preparation, characterization, and adsorption kinetics of graphene oxide/chitosan/carboxymethyl cellulose composites for the removal of environmentally relevant toxic metals. Int. J. Biol. Macromol..

[52] Ma M., Ke X., Wang T., Li J., Ye H. (2024). A novel double-network hydrogel made from electrolytic manganese slag and polyacrylic acid-polyacrylamide for removal of heavy metals in wastewater. J. Hazard. Mater..

[53] Gokmen F.O., Yaman E., Temel S. (2021). Eco-friendly polyacrylic acid based porous hydrogel for heavy metal ions adsorption: Characterization, adsorption behavior, thermodynamic and reusability studies. Microchem. J..

[54] Mishra A., Nath A., Pande P.P., Shankar R. (2021). Treatment of gray wastewater and heavy metal removal from aqueous medium using hydrogels based on novel crosslinkers. J. Appl. Polym. Sci..

[55] Wang X., Li X., Peng L., Han S., Hao C., Jiang C., Wang H., Fan X. (2021). Effective removal of heavy metals from water using porous lignin-based adsorbents. Chemosphere.

[56] Oliveira I.E., Silva R.M., Girao A.V., Faria J.L., Silva C.G., Silva R.F. (2020). Facile Preparation of ZnO/CNTs Nanocomposites via ALD for Photocatalysis Applications. Eur. J. Inorg. Chem..

[57] Russier J., Menard-Moyon C., Venturelli E., Gravel E., Marcolongo G., Meneghetti M., Doris E., Bianco A. (2011). Oxidative biodegradation of single- and multi-walled carbon nanotubes. Nanoscale.

[58] Temel N.K., Sertakan K.S. (2024). Comparison of the effect of various carbon-based nanomaterials for the removal of Cu^2+^ ions from aqueous solutions. Chem. Pap..

[59] Cheng Z., Lu Y., An J., Zhang H., Li S. (2022). Multi-scale reinforcement of multi-walled carbon nanotubes/polyvinyl alcohol fibers on lightweight engineered geopolymer composites. J. Build. Eng..

[60] Kang Z., Jia X., Ma X., Wen D., Carniato F. (2023). Modelling and Prediction of Fe/MWCNT Nanocomposites for Hexavalent Chromium Reduction. Processes.

[61] Thakur S., Arotiba O.A. (2018). Synthesis, swelling and adsorption studies of a pH-responsive sodium alginate-poly(acrylic acid) superabsorbent hydrogel. Polym. Bull..

[62] Thakur S., Pandey S., Arotiba O.A. (2016). Development of a sodium alginate-based organic/inorganic superabsorbent composite hydrogel for adsorption of methylene blue. Carbohydr. Polym..

[63] Mozaffari T., Vanashi A.K., Ghasemzadeh H. (2021). Nanocomposite hydrogel based on sodium alginate, poly (acrylic acid), and tetraamminecopper (II) sulfate as an efficient dye adsorbent. Carbohydr. Polym..

[64] Liu Y., Zhang L., Tang Y., Zhu L. (2021). Study on the Preparation and Adsorption Properties of Sodium Alginate Graft Polyacrylic Acid/Graphite Oxide Composite Hydrogel. Polym. Sci. Ser. A.

[65] Xie Q., Zou Y., Wang Y., Wang H., Du Z., Cheng X. (2022). Mechanically robust sodium alginate/cellulose nanofibers/polyethyleneimine composite aerogel for effective removal of hexavalent chromium and anionic dyes. Polym. Eng. Sci..

[66] Zhang X.M., Yang X.-L., Wang B. (2022). Self-healing and wearable conductive hydrogels with dynamic physically crosslinked structure. J. Mater. Sci.-Mater. Electron..

[67] Mo Y., Wang S., Vincent T., Desbrieres J., Faur C., Guibal E. (2019). New highly-percolating alginate-PEI membranes for efficient recovery of chromium from aqueous solutions. Carbohydr. Polym..

[68] Eltaweil A.S., Elgarhy G.S., El-Subruiti G.M., Omer A.M. (2020). Carboxymethyl cellulose/carboxylated graphene oxide composite microbeads for efficient adsorption of cationic methylene blue dye. Int. J. Biol. Macromol..

[69] Zhang W., Hu L., Hu S., Liu Y. (2019). Optimized synthesis of novel hydrogel for the adsorption of copper and cobalt ions in wastewater. RSC Adv..

[70] Cheng J., Shan G., Pan P. (2015). Temperature and pH-dependent swelling and copper(II) adsorption of poly(N-isopropylacrylamide) copolymer hydrogel. RSC Adv..

[71] Ngah W.S.W., Fatinathan S. (2010). Adsorption characterization of Pb(II) and Cu(II) ions onto chitosan-tripolyphosphate beads: Kinetic, equilibrium and thermodynamic studies. J. Environ. Manag..

[72] Xiong Q., Zhang F. (2022). Study on the Performance of Composite Adsorption of Cu^2+^ by Chitosan/β-Cyclodextrin Cross-Linked Zeolite. Sustainability.

[73] Yin K., Wu J., Deng Q., Wu Z., Wu T., Luo Z., Jiang J., Duan J.-A. (2022). Tailoring micro/nanostructured porous polytetrafluoroethylene surfaces for dual-reversible transition of wettability and transmittance. Chem. Eng. J..

[74] Li R., Wang J.J., Zhang Z., Awasthi M.K., Du D., Dang P., Huan Q., Zhang Y., Wang L. (2018). Recovery of phosphate and dissolved organic matter from aqueous solution using a novel CaO-MgO hybrid carbon composite and its feasibility in phosphorus recycling. Sci. Total Environ..

[75] Ha H.T., Huong N.T., Dan L.L., Tung N.D., Trung V.B., Minh T.D. (2020). Removal of Heavy Metal Ion Using Polymer-Functionalized Activated Carbon: Aspects of Environmental Economic and Chemistry Education. J. Anal. Methods Chem..

[76] Mu’azu N.D., Al-Yahya M., Al-Haj-Ali A.M., Abdel-Magid I.M. (2016). Specific energy consumption reduction during pulsed electrochemical oxidation of phenol using graphite electrodes. J. Environ. Chem. Eng..

[77] Das R., Giri S., Muliwa A.M., Maity A. (2017). High-Performance Hg(II) Removal Using Thiol-Functionalized Polypyrrole (PPy/MAA) Composite and Effective Catalytic Activity of Hg(II)-Adsorbed Waste Material. ACS Sustain. Chem. Eng..

[78] Paulino A.T., Guilherme M.R., Reis A.V., Tambourgi E.B., Nozaki J., Muniz E.C. (2007). Capacity of adsorption of Pb^2+^ and Ni^2+^ from aqueous solutions by chitosan produced from silkworm chrysalides in different degrees of deacetylation. J. Hazard. Mater..

[79] Xu Q., Xu Y., Xue J., Zhu F., Zhong Z., Liu R. (2021). An innovative alcohol-solution combustion-calcination process for the fabrication of NiFe_2_O_4_ nanorods and their adsorption characteristics of methyl blue in aqueous solution. Mater. Res. Express.

[80] Nodeh H.R., Kamboh M.A., Ibrahim W.A.W., Jume B.H., Sereshti H., Sanagi M.M. (2019). Equilibrium, kinetic and thermodynamic study of pesticides removal from water using novel glucamine-calix [4]arene functionalized magnetic graphene oxide. Environ. Sci.-Proc. Imp..

[81] Gunay A., Arslankaya E., Tosun I. (2007). Lead removal from aqueous solution by natural and pretreated clinoptilolite:: Adsorption equilibrium and kinetics. J. Hazard. Mater..

[82] Wang M., Li X., Hua W., Deng L., Li P., Zhang T., Wang X. (2018). Superelastic three-dimensional nanofiber-reconfigured spongy hydrogels with superior adsorption of lanthanide ions and photoluminescence. Chem. Eng. J..

[83] Papageorgiou S.K., Kouvelos E.P., Favvas E.P., Sapalidis A.A., Romanos G.E., Katsaros F.K. (2010). Metal-carboxylate interactions in metal-alginate complexes studied with FTIR spectroscopy. Carbohydr. Res..

[84] Juang R.S., Chen M.L. (1997). Application of the Elovich equation to the kinetics of metal sorption with solvent-impregnated resins. Ind. Eng. Chem. Res..

[85] Nie L., Chang P., Liang S., Hu K., Hua D., Liu S., Sun J., Sun M., Wang T., Okoro O.V. (2021). Polyphenol rich green tea waste hydrogel for removal of copper and chromium ions from aqueous solution. Clean. Eng. Technol..

[86] Tejada-Tovar C., Villabona-Ortiz A., Dario Gonzalez-Delgado A. (2021). Adsorption of Azo-Anionic Dyes in a Solution Using Modified Coconut (*Cocos nucifera*) Mesocarp: Kinetic and Equilibrium Study. Water.

[87] Yao L., Hong C., Qi Y.X., Wu L. (2025). Effective Co^2+^ removal from aqueous solution and industrial wastewater using ZIF-7 and MnFe_2_O_4_-modified walnut shell biochar composite. Sep. Purif. Technol..

[88] Pathania D., Sharma S., Singh P. (2017). Removal of methylene blue by adsorption onto activated carbon developed from Ficus carica bast. Arabian J. Chem..

[89] Liu Y., Kang Y., Mu B., Wang A. (2014). Attapulgite/bentonite interactions for methylene blue adsorption characteristics from aqueous solution. Chem. Eng. J..

[90] Uysal M., Ar I. (2007). Removal of Cr(VI) from industrial wastewaters by adsorption—Part I: Determination of optimum conditions. J. Hazard. Mater..

[91] Tempkin M.J., Pyzhev V. (1940). Recent modifications to Langmuir isotherms. Acta Physicochim.URSS.

[92] Marhoon A.A., Hasbullah S.A., Asikin-Mijan N., Mokhtar W. (2024). Adsorption of metal porphyrins using chitosan/zeolite-X composite as an efficient demetallization agent for crude oil: Isotherm, kinetic, and thermodynamic studies. Int. J. Biol. Macromol..

[93] Sharipova A.A., Aidarova S.B., Bekturganova N.Y., Tleuova A., Kerimkulova M., Yessimova O., Kairaliyeva T., Lygina O., Lyubchik S., Miller R. (2017). Triclosan adsorption from model system by mineral sorbent diatomite. Colloids Surf. A.

[94] Eftekhari M., Gheibi M., Azizi-Toupkanloo H., Hossein-Abadi Z., Khraisheh M., Fathollahi-Fard A.M., Tian G. (2021). Statistical optimization, soft computing prediction, mechanistic and empirical evaluation for fundamental appraisal of copper, lead and malachite green adsorption. J. Ind. Inf. Integr..

